# Carbon Nanomaterials With Hollow Structures: A Mini-Review

**DOI:** 10.3389/fchem.2021.668336

**Published:** 2021-03-30

**Authors:** Fan Liu, Yu Cheng, Junchao Tan, Jiantong Li, Haoyan Cheng, Hao Hu, Chunya Du, Shuang Zhao, Yan Yan, Mingkai Liu

**Affiliations:** ^1^Jiangsu Key Laboratory of Green Synthetic Chemistry for Functional Materials, School of Chemistry & Materials Science, Jiangsu Normal University, Xuzhou, China; ^2^Hubei Key Laboratory of Polymer Materials, Ministry-of-Education Key Laboratory for the Green Preparation and Application of Functional Materials, School of Materials Science & Engineering, Hubei University, Wuhan, China; ^3^Henan Engineering Laboratory of Flame-Retardant and Functional Materials, College of Chemistry and Chemical Engineering, Henan University, Kaifeng, China; ^4^Collaborative Innovation Center of Nonferrous Metals, School of Materials Science and Engineering, Henan University of Science and Technology, Luoyang, China

**Keywords:** carbon materials, nanostructures, hollow morphology, synthesis strategies, structural information

## Abstract

Carbon nanomaterials with high electrical conductivity, good chemical, and mechanical stability have attracted increasing attentions and shown wide applications in recent years. In particularly, hollow carbon nanomaterials, which possess ultrahigh specific surface area, large surface-to-volume ratios, and controllable pore size distribution, will benefit to provide abundant active sites, and mass loading vacancy, accelerate electron/ion transfer as well as contribute to the specific density of energy storage systems. In this mini-review, we summarize the recent progresses of hollow carbon nanomaterials by focusing on the synthesis approaches and corresponding nanostructures, including template-free and hard-template carbon hollow structures, metal organic framework-based hollow carbon structures, bowl-like and cage-like structures, as well as hollow fibers. The design and synthesis strategies of these hollow carbon nanomaterials have been systematically discussed. Finally, the emerging challenges and future prospective for developing advanced hollow carbon structures were outlined.

## Introduction

Carbon, one of the most important elements in nature, has been utilized in human civilization for more than 3,000 years (Hu et al., 2010). In the past decades, three significant breakthroughs mark that carbon based materials have entered the nano era: (1) In 1985, C60 namely fullerene was exploited by Kroto et al. (1985); (2) Iijima (1991) reported carbon nanotube in 1991; (3) In 2004, graphene was developed by Novoselov et al. (2004). Since then, carbon nanomaterials have become a hotspot, hence, various novel nanostructures and synthesis approaches have been developed (Wang Q. et al., 2017; Li et al., 2018; Zhang P. et al., 2018; Liu M. et al., 2019; Sun et al., 2019a; Zhan et al., 2019; Shen et al., 2020; Yan et al., 2020; Yang et al., 2020).

**Figure 1:**
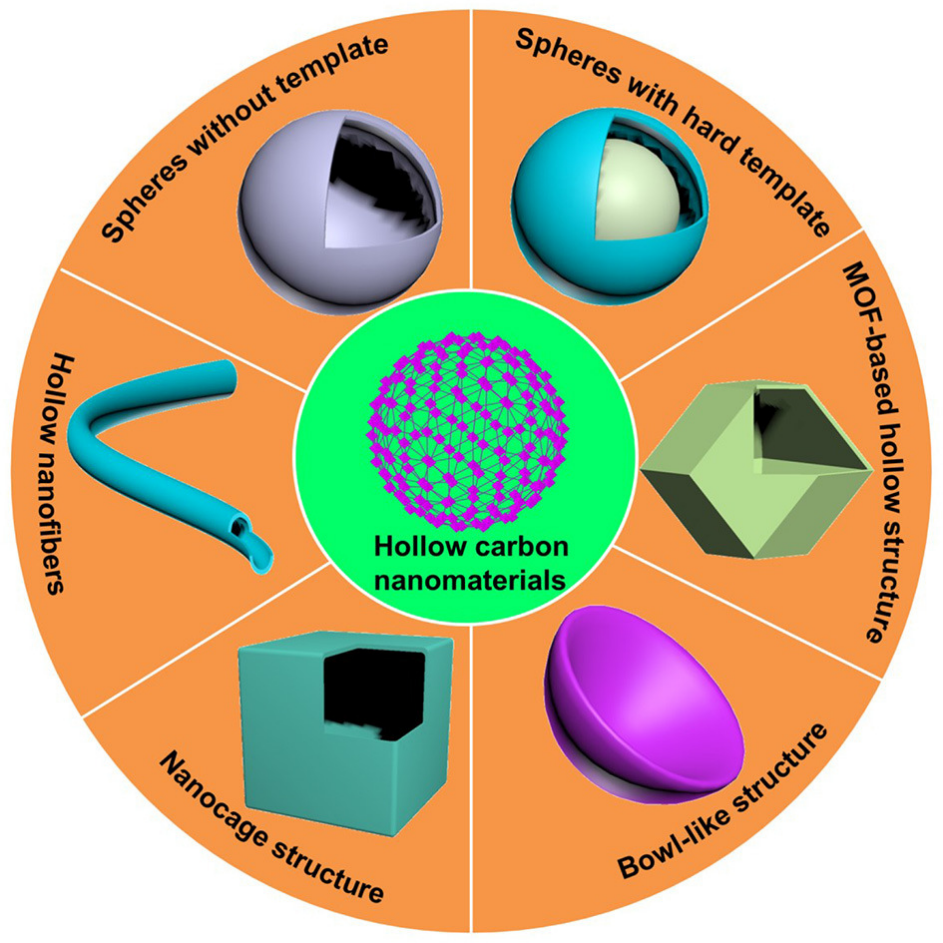
Schematic illustration of various carbon nanomaterials with hollow structures.

Recently, carbon-based nanomaterials have been widely developed and been used in many fields such as energy storage and conversion, photocatalysis, electrocatalysis, gas, and water treatment systems and biomedicine (Liu et al., 2017a,b, 2018; Yang et al., 2018; Liu Y. Q. et al., 2019; Sun et al., 2019b,c; Zhang and Lou, 2019; Guo et al., 2020; Wu C. et al., 2020; Yuan et al., 2020a,c,d). Among various carbon nanomaterials, hollow carbon nanostructures (HCNs) (Figure 1) have attracted considerable interests due to their high thermal stability, strong electron transport ability, large specific surface area, plentiful exposed active sites, and flexible shape and structure (Wen et al., 2007; Guo et al., 2019; Chen et al., 2020; Gao et al., 2020; Wang et al., 2020; Yuan et al., 2020b). A large number of studies have shown that HCNs exhibit excellent performance for energy, catalysis, electronics, biomedical, and so on in terms of their unique hollow structures.

In this mini-review, we summarize the recent advances of HCNs by focusing on the synthesis approaches and corresponding nanostructures, including hollow carbon spheres based on template-free and hard-template methods, metal organic framework-derived HCNs, bowl-like, cage-like, and fiber-shaped HCNs. The emerging challenges and future prospective for developing advanced hollow carbon structures were also outlined. We believe that this mini-review could offer some new insights and inspire extensive interests to accelerate and explore the innovations of HCNs.

## Hollow Carbon Based on Template-Free Approach

Template-free method, which also termed self-template method, is regarded as a facile and one-step strategy for the synthesis of HCNs. Usually, template-free method involves Kirkendall effect, Ostwald ripening, ion exchange, and selective etching (Zhang and Lou, 2019).

In 2001, Wang et al. first reported a facile hydrothermal route to prepare monodispersed hard carbon spherules. Sugar was selected as precursor, and hydrothermal treated for 5 h at 190°C, and followed by a carbonizing process, the carbon spheres were obtained (Wang et al., 2001). The as-prepared carbon sphere exhibited a specific surface area of 400 m^2^/g and lithium storage capacity up to 430 mAh/g, which undoubtedly opened the door for practical application of carbon materials in energy storage. After that, X. Sun and Y. Li studied the mechanism of preparing carbon spheres from glucose by hydrothermal method, and further developed a general synthesis strategy of hybrid, hollow, or porous carbon spheres (Sun and Li, 2004). To date, hydrothermal or solvothermal carbonization method has been developed into a classical strategy, by which numerous hollow carbon spheres were successfully synthesized (Han et al., 2011; Liang et al., 2015; Chen et al., 2018; Wang et al., 2019).

**Figure 2:**
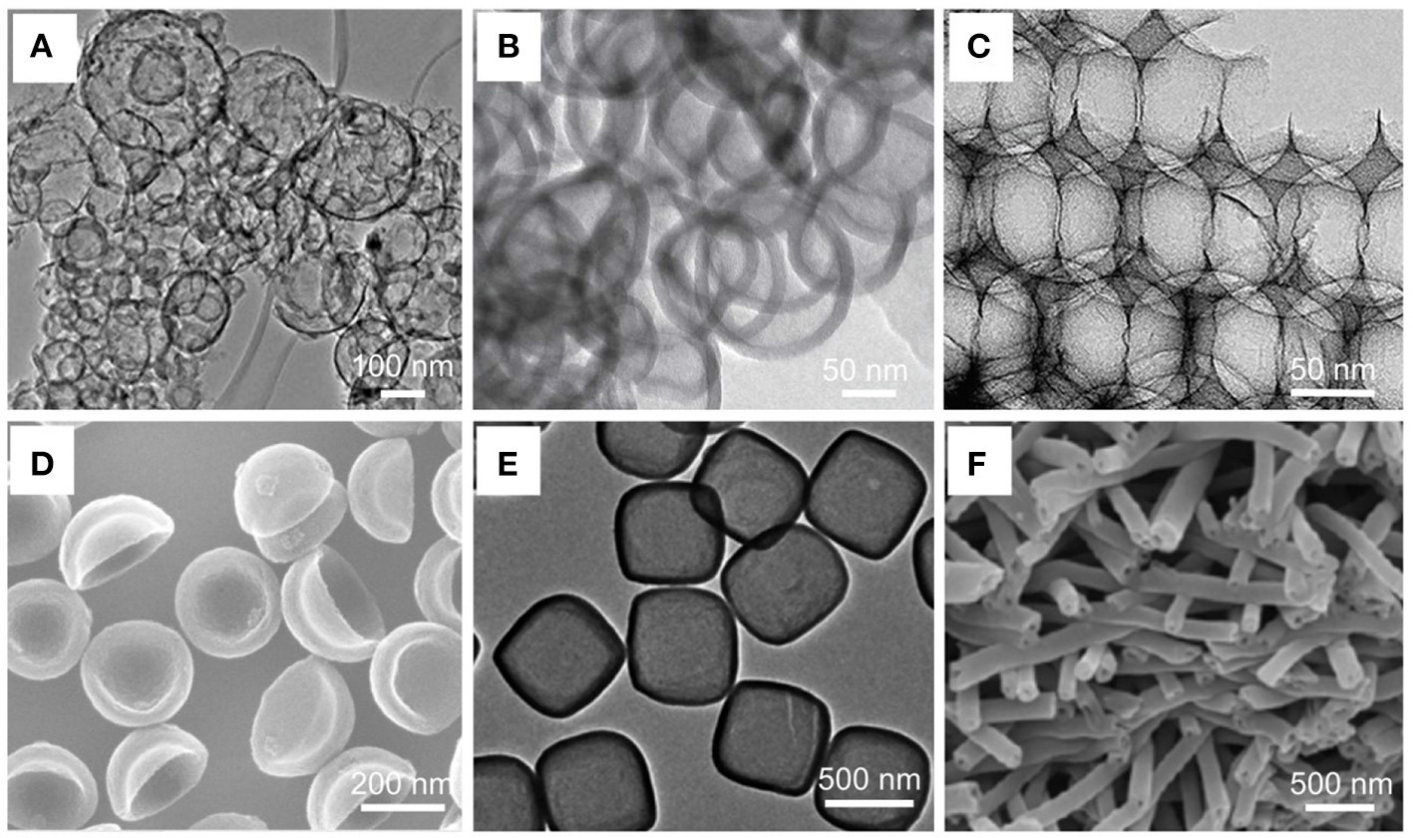
**(A)** TEM image of template-free synthesized carbon hollow spheres (CHS). Natarajan et al. (2019) with permission from Royal Society of Chemistry. **(B)** TEM image of hollow carbon nanospheres with latex templates. Tang et al. (2012) with permission from Wiley-VCH. **(C)** TEM image of bicontinuous hierarchical porous carbon (BHPC-950) after hydrofluoric acid etching. Yang et al. (2017) with permission from Wiley-VCH. **(D)** SEM image of N-doped hollow porous carbon bowls (N-HPCB). Pei et al. (2016) with permission from Wiley-VCH. **(E)** TEM image of carbon nanoboxes. Yu et al. (2015) with permission from Wiley-VCH. **(F)** SEM image of N-doped hollow carbon nanofibers. Ramakrishnan et al. (2015) with permission from Royal Society of Chemistry.

In recent years, a great many of new strategies have been developed. For instance, a facile one-step carbonization process to prepare hollow carbon spheres with different sizes from 100 to 400 nm, was presented by Natarajan and co-workers. Polypropylene (PP) and polyethylene (PE), which recovered from spent lithium-ion batteries, were heated at 800°C for 2 h. After cooling and washing with benzene, the product with specific surface area and total pore volume of 402 m^2^/g and 0.30 cm^3^/g was obtained (Figure 2A) (Natarajan et al., 2019). In addition, Sun and co-authors synthesized N-doped hollow carbon spheres by stepwise polymerizing and carbonizing procedure. Industrialized monomers, pyromellitic dianhydride (PMDA) and 4, 4-oxydianiline (ODA), were used as raw materials, and stepwise polymerized at ambient temperature, the obtained homopolymer self-assembled into poly (amic acid) (PAA) vesicles. After carbonizing at 800°C for 3 h, PAA vesicles transformed into hollow carbon spheres (Sun et al., 2016). Similarly, initiated by ammonium sulfate, 2, 6-Diaminopyridine polymerized and then heated at 950°C for 1 h, N, S co-doped hollow carbon spheres were prepared (Zhang X. et al., 2019).

## Hollow Carbon With Hard–Template Method

As an effective strategy, hard-template method, in which the materials with low price and easily controlled are used as templates, followed by being removed with physical or chemical procedure, is widely applied to synthesize HCNs. In general, hard-template method involves four steps. Firstly, a rigid solid template is prepared with specific morphology. Then, the surface of as-prepared template was modified or functionalized in order to increasing absorption ability. Furthermore, carbon precursor (i.e., dopamine, PAN, PAA, P123, P127) was coated on the surface of as-prepared template. Finally, as-prepared template was removed by solution etching or high temperature degradation.

Since hard-template method was discovered in 1999, numerous nanostructures have been prepared. Yoon and co-authors synthesized hollow core/mesoporous shell for the first time by using silica spheres as templates (Yoon et al., 2002). Subsequently Joo et al. developed a facile hydrothermal approach to prepare hollow carbon sphere by using silica as template and sucrose as carbon source. First, silica particles were prepared by a classical Stöber approach and impregnated with AlCl_3_ aqueous solution. Next, sucrose was added as carbon precursor and treated by a benign hydrothermal process. After annealing (850°C) and etching (HF) treatment, the hollow carbon spheres were obtained (with specific surface of 788 cm^2^/g, pore volume of 1.15 cm^3^/g) (Joo et al., 2008). In addition, Tang and co-workers demonstrated that hollow carbon spheres can be prepared via a facile hydrothermal carbonization method. Polystyrene latexes and glucose were mixed and hydrothermally treated at 180°C for 20 h, and subsequently heated at 1000°C (Figure 2B) (Tang et al., 2012).

## Metal Orginic Frameworks Derived Hollow Carbon Nanomaterials

Since the metal organic frameworks (MOFs) were synthesized in the 1990s, they have been widely used in adsorption, separation, catalysis, energy storage, pharmaceutical, and other fields due to their large specific surface area, porosity, convenient synthesis, good thermal stability, variable skeleton size, and chemical modification (Ren et al., 2018; Sun et al., 2020a,b). Selecting the precursors and calcination conditions of MOFs is an effective strategy to prepare new carbon nanomaterials with controllable size, shape, and composition. In 2008, Liu and co-authors reported that MOF was applied as template to synthesize porous carbon nanomaterials (Liu et al., 2008). MOF-5 and furfuryl alcohol were used as template and carbon source, respectively, followed by dynamic vacuum (200°C, 24 h) and carbonizing process (1000°C, 8 h, N_2_), porous carbon with high specific surface area (2872 m^2^/g) was obtained. Since then, various HCNs have been prepared and applied in many fields. For instance, through a controlled etching approach, novel nanosize monocrystalline hollow MOF nanobubbles with a uniform size of <100 nm and a thin shell of around 10 nm were prepared (Zhang et al., 2017). Moreover, Yang and co-workers reported a dual-template route to nitrogen-rich porous carbon. Typically, 3D ordered SiO_2_ infiltrated into ZIF-8, then heated at 800–1000°C and etched by hydrofluoric acid, the obtained product exhibited ultralarge surface area of 2546 m^2^/g and ultrahigh total pore volume of 13.42 cm^3^/g (Figure 2C) (Yang et al., 2017).

On the other hand, combining MOFs with various functional materials is also an effective and feasible strategy. In 2012, Jahan and co-authors used reduced graphene oxide (rGO) sheets, which were functionalized with pyridine ligands on either side of the surface, acting as pillars connecting metalloporphyrin nodes to form a hybrid grapheme-MOF framework. Their excellent work demonstrated that the addition of functionalized rGO can influence the crystallization process of MOF and improve the electrocatalytic properties of the composites (Jahan et al., 2012). Additionally, hybridizing with carbon nanotubes is a widely adopted strategy in recent years. By dispersing and penetrating carbon nanotubes with MOF precursors, a large number of hybrid structures, such as multi-walled carbon nanotube/zeolitic ZIF-8 composite (MWNT@ZIF) (Yue et al., 2014), interpenetrated and self-standing conductive framework (ISCF) were successfully prepared (Liu Y. et al., 2017). In 2018, Zhang and co-authors reported a 3D porous MOF@CNT hybrid structure through a facile impregnation and solvothermal reaction approach. A carbon tube sponge which used as template was produced first, and then immersed into precursor solution. After solvothermal treatment, the ZIF-8 was *in situ* synthesized and MOF@CNT hybrid was obtained (Zhang H. et al., 2018).

## Hollow Bowl-Like Carbon Nanomaterials

Hollow bowl-like carbon nanomaterials have attracted great interests due to their excellent properties, such as large surface area, tunable pore sizes, high pore volume, high packing density, high electrical conduction (Liang et al., 2020). Just as their hollow carbon spheres counterparts, hollow bowl-like carbon nanomaterials can be prepared via template-assisted method or template-free method as mentioned above.

In 2016, Pei used SiO_2_ and polybenzoxazine as hard template and carbon precursor, respectively, and N-Doped hollow porous carbon bowls (N-HPCB) were synthesized with the treatments of carbonizing and etching (Figure 2D). The as-prepared N-HPCB exhibited a high specific surface area up to 2161 m^2^/g and pore volume of 1.5 m^3^/g (Pei et al., 2016). Without carbonization, Gao and co-authors developed a generalized strategy for the synthesis of “dual carbon”-protected bowl-like hollow particles. Similarly, SiO_2_ and resorcinol formaldehyde (RF) were used as hard template and carbon source, respectively. A low-temperature refluxing procedure and a vapor-phase process were utilized during the preparation process (Gao et al., 2019). Interestingly, many facile strategies have been developed that hollow bowl-like carbon can be obtained just by tuning mass ratio of RF and tetraethyl orthosilicate (TEOS) (Fei et al., 2020; Yi et al., 2020).

As known, compared with its counterpart, template-free method requires fewer steps and cause less waste. Hence, scientists have focused their interests on synthesizing hollow bowl-like carbon materials with template-free method. With a facile sulfuric acid treatment and drying route, Liang and co-workers prepared hollow bowl-like carbon with specific surface area of 103.8 m^2^/g (Liang et al., 2014). In addition, Duan and co-authors prepared hollow bowl-like carbon supported AuPd with an average size of 175 nm by traditional hydrothermal carbonization (Duan et al., 2020).

## Carbon Nanocages

Unlike sphere-shaped carbon nanomaterials, mass production of nanocages from carbon materials is still a great challenge. Template-assisted method is an effective strategy for the synthesis of carbon based nanocages. Xie et al. reported a facile route to prepare carbon nanocages by using MgO and benzene as template and carbon source, respectively (Xie et al., 2012). Typically, basic magnesium carbonate was heated in a tubular furnace, and then benzene was added into the tubular furnace, followed by treated with hydrochloric acid solution, the carbon nanocages with specific surface area up to 2053 m^2^/g were obtained. Similarly, Zang and co-workers prepared carbon nanocages by using SiO_2_ and resorcinol formaldehyde resin as template and carbon source, respectively (Zang et al., 2020). In addition, Fe_3_O_4_, TiO_2_, and CaO also were used as templates to prepare carbon nanocages (Wu Q. et al., 2020). Such as, novel N-doped carbon nanoboxes were synthesized with Fe_2_O_3_ nanocubes as the template, and a thin layer of polydopamine (PDA) were coated on their surface, followed by carbonization process (500°C, 3 h) and being etched by hydrochloric acid, which resulted in the preparation of N-doped carbon nanoboxes (Figure 2E) (Yu et al., 2015).

More recently, pyrolysis has become a new strategy to prepare carbon nanocages. In 2019, Zhang and co-workers successfully synthesized carbon nanocages by the pyrolysis (600°C, 3 h) of PE and magnesium powder (Zhang Y. et al., 2019). Moreover, Wang and co-workers prepared N-doped carbon nanocages by a spay pyrolysis of pyridine (C_5_H_5_N) and pentacarbonyl [Fe(CO)_5_] at 700 and 1000°C (Wang et al., 2014).

## Hollow Carbon Fibers

As early as 1997, by introducing alumina membrane as template, Hulteen and co-authors prepared hollow carbon fibers (Hulteen et al., 1997). Typically, porous alumina membrane was immersed in acrylonitrile solution, then the polymerization reaction was initiated by adding 1, 1′-azobis(cyclohexane carbonitrile), resulting in the formation of PAN/alumina composite. Similarly, in 2011, Zheng and co-workers reported a hollow carbon nanofiber-encapsulated sulfur electrode structure (Zheng et al., 2011). Anodic aluminum oxide (AAO) membrane and polystyrene (PS) were chosen as template and carbon source, respectively, through a carbonization process (750°C,4 h), carbon coated AAO membranes were prepared, followed by removing the AAO templates, hollow carbon fibers of diameters range between 200 and 300 nm were obtained. Interestingly, biomass material, such as crab shell can also be used as template to prepare hollow carbon fibers (Liu et al., 2010).

On the other hand, electrospinning is also widely used for the synthesis of hollow carbon fibers. Larsen and co-workers reported a facile coaxial route for the fabrication of hollow nanofibers. TEOS and olive oil were used as outside and inner nanojet liquid, respectively. And they were injected into two coaxial capillaries with different diameters. After co-electrospinning process, hollow nanofibers were obtained (Loscertales et al., 2004). Similarly, Xia and Li fabricated hollow nanofibers by using Poly(vinyl pyrrolidone) (PVP) and Ti(OiPr)_4_ as the core and shell materials, followed by co-electrospinning and carbonizing process (500°C, 1 h) (Li and Xia, 2004). Since then, various materials such as, polyacrylonitrile (PAN), poly(styrene-co-acrylonitrile) (SAN) (Le et al., 2016), poly (methyl methacrylate) (PMMA) have been used to synthesize hollow carbon fibers (Wang Y. et al., 2017). For instance, Ramakrishnan and co-workers reported a facile coaxial electrospinning approach to prepare hollow carbon fibers. PAN and PVP were used as carbon source and sacrificial material, respectively. After coaxial electrospinning and carbonization process (800°C,1 h), the as-prepared hollow carbon fibers exhibited surface area of 557 m^2^/g and ultrahigh total pore volume of 0.5681 cm^3^/g (Figure 2F) (Ramakrishnan et al., 2015). In addition, with the development of nanotechnology, combing electrospinning with other technologies has become a new research hotspot in recent years. For example, by combing electrospinning with template-assisted method, Sun et al. prepared fantastic bamboo-like hollow carbon fibers (Sun et al., 2015). Firstly, PAN and TEOS were used as precursors to prepare white nanofibers by electrospinning. Then bamboo-like hollow carbon fibers were obtained after carbonization treatment followed by removing SiO_2_ template in hydrofluoric acid.

## Discussion

In summary, recent advances in synthesis of carbon nanomaterials with hollow structures are reviewed and discussed. The synthesis methods and applications of hollow carbon spheres, metal organic framework-derived carbon structures, bowl-like, cage-like, and carbon fibers hollow structures are presented. Although great progress has been made in this field, its synthesis, application and precise structural adjusting of HCNs are still facing great challenges. (1) Although template-free method is simple, the dynamics process of structure formation is not clarified. (2) Hard-template method is one of the most effective methods for synthesizing HCNs. However, sodium hydroxide or hydrofluoric acids are inevitably used in the process of template etching, especially for SiO_2_ template, which not only pollutes the environment, but also increase the cost. Therefore, developing a novel template which could be removed under mild conditions is becoming to be the emphasis of research. (3) A large amount of metal organic frameworks have been reported over the years. Nonetheless, only few of them (i. e., MOF-5, ZIF-8, ZIF-67) could be derived to hollow carbon nanomaterials, due to their poor thermal structure stability. It is apparent that exploiting a series of ligands for high stability MOF or developing new carbonization approaches will broad their application on HCNs. (4) Compared with single layer hollow carbon nanomaterials (i.e., bow-like carbon nanomaterials, carbon nanocages, and hollow carbon fiber), multilayer hollow structure, or hollow hierarchical structure, which could increase the specific surface area, modify properties of different layer, enhance the connection of each hollow structure, will be the key point in the development of hollow carbon nanomaterials.

Hollow carbon nanomaterials undoubtedly become a hot spot of novel materials research due to their unique structures with high specific area, rich exposed active sites, and mass loading vacancies. It is believed that with the combined efforts in traditional methods and in-depth kinetic analysis, more strategies will be realized in building unified size, stable, environmentally friendly, and low cost hollow carbon nanomaterials. These insightful ideas, raised during the exploration, will eventually benefit the understanding and development of conventional hollow structure, as well as other nanomaterials based on hollow structure.

## Author Contributions

FL and JT wrote the manuscript. FL and YC collected and read papers and contributed to the Discussion section. JL and SZ contributed to the paper design and refine. HC, HH, and CD contributed to the proofreading of the paper. ML and YY revised and approved the manuscript. All the authors collected and read papers and contributed to paper writing.

## Conflict of Interest

The authors declare that the research was conducted in the absence of any commercial or financial relationships that could be construed as a potential conflict of interest.
